# Effect of recurrent severe insulin-induced hypoglycemia on the cognitive function and brain oxidative status in the rats

**DOI:** 10.1186/s13098-024-01410-z

**Published:** 2024-07-15

**Authors:** Mahvash Nikpendar, Mohammad Javanbakht, Hamidreza Moosavian, Sepideh Sajjadi, Yalda Nilipour, Toktam Moosavian, Mahsa Fazli

**Affiliations:** 1 Brain and Spinal Injury Research Center, Neuroscience Institute, Tehran University of Medical Sciences, Tehran, Iran; 2 Nephrology and Urology Research Center, Clinical Science Institute, Baqiyatallah University of Medical Sciences, Tehran, Iran; 3 Department of Clinical Pathology, Faculty of Veterinary Medicine, University of Tehran, Tehran, Iran; 4 Pediatric Pathology Research Center, Research Institute for Children Health, Shahid Beheshti University of Medical Sciences, Tehran, Iran; 5 Pediatric Neurology Department, Loghman Hakim Hospital, Shahidbeheshti University of Medical Sciences, Tehran, Iran; 6 Department of Biology, Faculty of Basic Science, Islamic Azad University, Tehran, Iran

**Keywords:** Insulin, Hypoglycemia, Cognitive function, Hippocampus, Oxidative stress

## Abstract

**Background:**

Episodes of recurrent or severe hypoglycemia can occur in patients with diabetes mellitus, insulinoma, neonatal hypoglycemia, and medication errors. However, little is known about the short-term and long-term effects of repeated episodes of acute severe hypoglycemia on the brain, particularly in relation to hippocampal damage and cognitive dysfunction.

**Methods:**

Thirty-six wistar rats were randomly assigned to either the experimental or control group. The rats were exposed to severe hypoglycemia, and assessments were conducted to evaluate oxidative stress in brain tissue, cognitive function using the Morris water maze test, as well as histopathology and immunohistochemistry studies. The clinical and histopathological evaluations were conducted in the short-term and long-term.

**Results:**

The mortality rate attributed to hypoglycemia was 34%, occurring either during hypoglycemia or within 24 h after induction. Out of the 14 rats monitored for 7 to 90 days following severe/recurrent hypoglycemia, all exhibited clinical symptoms, which mostly resolved within three days after the last hypoglycemic episode, except for three rats. Despite the decrease in catalase activity in the brain, the total antioxidant capacity following severe insulin-induced hypoglycemia increased. The histopathology findings revealed that the severity of the hippocampal damage was higher compared to the brain cortex 90 days after hypoglycemia. Memory impairments with neuron loss particularly pronounced in the dentate gyrus region of the hippocampus were observed in the rats with severe hypoglycemia. Additionally, there was an increase in reactive astrocytes indicated by GFAP immunoreactivity in the brain cortex and hippocampus.

**Conclusion:**

Recurrent episodes of severe hypoglycemia can lead to high mortality rates, memory impairments, and severe histopathological changes in the brain. While many histopathological and clinical changes improved after three months, it seems that the vulnerability of the hippocampus and the development of sustained changes in the hippocampus were greater and more severe compared to the brain cortex following severe and recurrent hypoglycemia. Furthermore, it does not appear that oxidative stress plays a central role in neuronal damage following severe insulin-induced hypoglycemia. Further research is necessary to assess the consequences of repeated hypoglycemic episodes on sustained damage across various brain regions.

**Supplementary Information:**

The online version contains supplementary material available at 10.1186/s13098-024-01410-z.

## Background

The brain, being a dynamic organ, exhibits elevated metabolic demands and despite its ability to derive energy from lactate, pyruvate, and ketone bodies, glucose stands out as the primary substrate for brain energy metabolism [1]. The brain accounts for 25% of the total body glucose consumption [1]. Additionally, astrocytes utilize glucose to synthesize glutamine, a precursor essential for neurotransmission in neurons [2].

Hypoglycemia, characterized by abnormally low blood glucose levels, poses significant risks of neuronal injury and can precipitate cognitive impairment while hastening the onset of dementia [3, 4]. The occurrence of hypoglycemia is notably frequent among diabetic individuals undergoing insulin or sulphonylurea therapy, representing a primary challenge in managing diabetes mellitus [3, 5]. Moreover, underlying conditions such as insulinoma and dysregulated secretion of glucagon and growth hormone are identified as causative factors for hypoglycemia [5, 6]. Clinically, hypoglycemia is categorized into asymptomatic, mildly symptomatic, moderate, or severe states. Mild hypoglycemia is characterized by blood glucose levels dropping to 70 − 50 mg/dl, with or without accompanying symptoms, whereas moderate and severe hypoglycemia manifest when blood glucose levels decline to 60–40 mg/dl and fall below 40 mg/dl, respectively [7]. On the other hand, neuroglycopenic symptoms arise from reduced brain glucose levels and encompass cognitive impairments such as confusion, blurred vision, dizziness, irritability, speech difficulties, faintness, drowsiness, thought processing challenges, seizures, and comatose states [8].

Notably, hypoglycemia emerges as a pivotal factor in the etiology of neurocognitive disorders such as Alzheimer’s disease [9]. Transient mild hypoglycemia can induce reversible cognitive dysfunction, whereas persistent or severe hypoglycemic episodes can lead to irreversible neurological damage, altering brain structure and cognitive function irreversibly [10].

Recurrent episodes of hypoglycemia in patients could induce the failure of the counterregulatory hormonal response, reduce the glucose levels that trigger the counterregulatory autonomic response during a subsequent hypoglycemic period, and increase the risk of severe hypoglycemia. Recurrent severe hypoglycemia and the failure of counter-regulatory hormones are the possible aetiologies in the onset of neuroglycopenia before the appearance of autonomic warning symptoms. This phenomenon is referred to as hypoglycemia unawareness and is commonly observed in individuals with type 1 diabetes mellitus and with less frequency in type 2 diabetes mellitus [11]. Hypoglycemia-associated autonomic failure (HAAF) is an aftermath of hypoglycemia unawareness and autonomic response impairment, substantially heightening the risk of severe hypoglycemia by as much as 25-fold, culminating potentially in coma, irreversible cerebral damage, and even fatality [8, 12, 13]. The nature and severity of cerebral impairment and underlying pathophysiological processes incited in hypoglycemic patients are diverse and primarily contingent upon blood glucose concentrations. Functional cerebral insufficiency, reduced cognitive function, aberrant conduct, coma, and seizures may manifest in patients experiencing moderate hypoglycemia (plasma glucose levels below 2.8 mmol/L (50 mg/dL)), while neuronal demise may ensue in cases of profound hypoglycemia (blood glucose levels below 1.1 mmol/L (20 mg/dL)) [12].

A panoply of mechanisms are speculated to underlie the pathogenesis in instances of hypoglycemia-induced functional brain failure and neuronal death, encompassing cerebral energy deprivation, glutamate release and neuronal glutamate receptor activation, poly(ADP-ribose) polymerase activation, mitochondrial permeability transition, neuronal zinc liberation, and reactive oxygen species generation [12, 14]. Notably, insulin therapy stands out as a primary and prevalent causative factor of hypoglycemia in diabetic patients [3].

Nevertheless, certain research findings suggest that insulin exhibits antioxidative properties and can mitigate oxidative stress responses [15]. Thus, it appears that insulin plays a dual role, acting as an antioxidant while potentially instigating oxidant stress in patients through hypoglycemia.

The present study delves into alterations in antioxidants and histopathological changes in the brain subsequent to high doses of insulin administration and severe hypoglycemia. Furthermore, this study scrutinizes the impact of this disorder on cognitive deficits in rat. Our findings aim to shed light on the relationship between recurrent severe hypoglycemia and the transient and permanent brain injuries.

## Materials and methods

### Experimental animal experimental design

One-month-old male wistar rats (200–210 g) were procured from the Pasteur Institute of Iran for the experimental study. The animals were housed in a controlled environment with specific conditions set at 22 ± 1 °C temperature and 50 ± 10% humidity. A 12-hour light-dark cycle was maintained, and the rats had ad libitum access to food and water. Following a week of acclimatization, the rats were randomly assigned into three batches, totaling 6 groups. Each batch consisted of control groups (CG, *n* = 5) and hypoglycemic groups (HG, *n* = 7).

The HG rats underwent induced recurrent severe hypoglycemia through the administration of high doses of short-acting insulin (Regular insulin, 40IU/kg, Exir Pharmaceutical Co. Iran) after an overnight fast of 8 h, over three consecutive days. Conversely, the CG groups received equivalent volumes of normal saline under identical conditions.

Regular monitoring of tail vein glucose levels was conducted using an Accu-Chek glucometer (Roche Diabetes Care Inc, Germany) at 30-minute intervals to maintain low blood glucose levels (Mean: 0.85-1 mmol/L) for 90 min. Administration of glucose (1 mg/kg, intraperitoneal) was utilized to halt hypoglycemia, followed by subsequent blood glucose assessments. Rats exceeding 1 mmol/L during the initial monitoring period or surpassing 7.5 mmol/L post-glucose injection were excluded from the study.

A total of 32 rats were subjected to hypoglycemia, of which 11 succumbed (11/32, 34%) post-modeling, and the remaining 21 rats were divided into three groups (*n* = 7). Also, a total of 15 rats were divided into three groups of 5 rats each and were considered as control.

Following 24 h of severe hypoglycemia induction, 12 rats (First batch, CG, *n* = 5; and HG, *n* = 7) were sacrificed for the evaluation of brain antioxidant capacity. On the seventh day post-hypoglycemia, another 12 rats (Second batch, CG, *n* = 5; and HG, *n* = 7) were sacrificed for histological and immunohistological examinations. The remaining rats (Third batch, CG, *n* = 5; and HG, *n* = 7) were subjected to cognitive function assessments using the Morris water maze test on the 60th day of severe hypoglycemia, followed by subsequent sacrifice for histological and immunohistological studies on the 90th day.

### Assessment of oxidative stress markers

For the evaluation of cerebral oxidative stress markers, a total of 12 Rats (First batch, CG, *n* = 5; and HG, *n* = 7) were humanely euthanized subsequent to 24 h following the onset of the third episode of hypoglycemia. Succinctly, the rats were ethically sacrificed via decapitation, whereby the cranium was promptly dissected to extract the brain tissue. These excised brains were expeditiously placed upon absorbent filter paper saturated with normal saline atop a chilled glass surface imbued with pulverized ice. Subsequently, the parietal cortex and hippocampus regions were meticulously excised, aliquoted into Eppendorf tubes, and cryogenically preserved at -80 °C until further analytical scrutiny.

Tissue homogenates derived from the parietal cortex and hippocampus were meticulously prepared to ascertain the collective antioxidant potential (as denoted by Total Antioxidant Capacity - TAC), catalase enzymatic activity, malondialdehyde (MDA) levels, and superoxide dismutase (SOD) content, employing a commercially sourced assay kit from Navand Salamat Company, based in Urmia, Iran. In a concise manner, 50 to 60 milligrams of cerebral tissue underwent a thorough cold PBS rinse prior to homogenization in 1000 µl of lysis buffer. The resultant homogenates were subsequently subjected to centrifugation (at 8000 revolutions per minute [rpm], for 15 min at 4 °C), following which the supernatant was meticulously harvested. Thereafter, the prescribed enzymatic reactions were meticulously executed within the supernatant, adhering precisely to the manufacturer’s stipulated guidelines for each diagnostic kit.

The optical densities corresponding to the enzymatic reactions were recorded using an ELISA plate reader sourced from KHB, located in China. Furthermore, the protein concentration within the samples was ascertained utilizing Bradford’s methodology (Zist Pazhohan Co, Tehran, Iran).

### Total antioxidant capacity

The cerebral TAC was assessed employing the method delineated by Benzie and Strain (1996) [16]. This approach quantifies TAC by gauging the antioxidant potential to convert ferric ions to ferrous ions under acidic conditions, resulting in the formation of a distinct Ferrous-tripyridyltriazine complex, amenable to spectrophotometric quantification. Within the confines of this investigation, a conglomeration totaling 1000 µl was concocted, comprised of 100 µl of supernatant and 900 µl of varied reagents from the kit, meticulously adhering to the manufacturer’s guidelines. Post a brief 5-minute incubation period at ambient temperature, the absorbance was scrutinized at a wavelength of 630 nm.

The TAC content of the sample was computed utilizing the established equation (y = 0.0002x + 0.4625; R² = 0.99), derived from the standards optical densities and concentrations. The results are delineated in µmol/mg protein units.

### Catalase

Catalase is a heme antioxidant enzyme ubiquitous in nearly all aerobically active cells, responsible for the conversion of the reactive oxygen species hydrogen peroxide into molecular oxygen and two water molecules.

The catalase activity was ascertained following the methodology outlined by Johansson and Borg (1988) [17]. In this method, catalase enzyme in the presence of hydrogen peroxide and methanol (as a hydrogen donor) produces a type of aldehyde (formaldehyde). The resulting formaldehyde can be measured in combination with chromogen.

In this particular investigation, a final reaction mixture of 240 µl was meticulously prepared, comprising 20 µl of supernatant and 220 µl of various reagents provided in the assay kit, meticulously following the manufacturer’s stipulations. The absorbance measurement was conducted using an ELISA reader set to 540 nm. The samples’ catalase activity was calculated utilizing the formaldehyde standard formula (y = 0.0006x + 0.0423; R^2^ = 0.99), derived from the standards ODs and concentrations. The results are denoted as nmol/min/mg of protein.

### Malondialdehyde

To evaluate the extent of MDA as a biomarker for lipid peroxidation, the thiobarbituric acid assay was utilized, using spectrophotometry with the Navand Salamat Kit. The same approach utilized for determining the TAC was applied for preparing both the working and stock standard solutions. Subsequently, the absorbance of the resulting solution was measured at a wavelength of 550 nm using a microplate reader. The results are expressed as µmol/mg protein.

### Superoxide dismutase

To measure SOD activity, the Navand Salamat Kit was employed. The experimental procedure hinges on the retardation of pyrogallol autoxidation reaction. Pyrogallol is a substance prone to oxidation in the presence of air under normal conditions. By maintaining a defined concentration of this substance, the half-life of its autoxidation is ascertained. In this reaction, upon containing a sample of unknown SOD concentration, the inhibition extent of pyrogallol autoxidation reaction is gauged at a fixed time point and juxtaposed against a control, thereby gauging the SOD concentration level in the tested sample. The technique employed for formulating the working and stock standard solutions mirrored that utilized for TAC measurement. Subsequently, the optical density of the samples was gauged at a wavelength of 405 nm using an ELISA reader. The results are depicted in International Units per milligram of protein (IU/mg protein).

### Intracardiac perfusion, brain slice preparation, and histopathology study

For the assessment of histopathological changes over short and long term periods, a total of 12 rats were included (second batch, CG, *n* = 5; and HG, *n* = 7) in 7 days, and 12 Rats (third batch, CG, *n* = 5; and HG, *n* = 7) were euthanized in 90 days after the induction of acute hypoglycemia.

Briefly, the rats were subjected to anesthesia through the intraperitoneal (i.p.) route using a combination of ketamine (80 mg/kg) and xylazine (8 mg/kg). Following the induction of terminal anesthesia, the animals underwent transcardial perfusion with saline solution, and then were further perfused with 4% paraformaldehyde as previously detailed in the literature [18]. After completing the perfusion, the rats were decapitated, and the cerebellum as well as the hippocampus were promptly excised. The extracted tissues were then immersed in a 10% neutral buffered formalin solution overnight, subsequently dehydrated in xylene, and finally embedded in paraffin. The paraffin-embedded tissue blocks were then sectioned to a thickness of 5 μm utilizing a rotary microtome. The obtained sections were stained with Hematoxylin and Eosin and subjected to histopathological examination.

### Immunohistochemistry staining, GFAP staining

Immunohistochemical staining for glial fibrillary acidic protein (GFAP) was conducted to visualize the reactive astrocytes in the brain tissue. Briefly, after careful washing of the sections in PBS, a reaction with 0.3% H202 was carried out to bleach endogenous peroxidase. Sections were then incubated in rabbit anti-GFAP (1:2.000; Z 334, DAKO), overnight, followed by rabbit anti-rat IgG conjugated to horseradish peroxidase (1:100; P 450, DAKO). The peroxidase reaction was visualized using diaminobenzidine (0.05%) and H202 (0.01%). Sections were floated onto gelatine-coated slides, cleared in ascending grades of alcohol and xylene, and then mounted in DePeX (Serva, Heidelberg, Germany). A modified semi-quantitative scoring system (scoring range 0–3) was applied to all tissues for GFAP immunoreactivity [19]. Briefly, the four-point ranking scale was based on a combination of cellular morphology and the overall density in the viewing field: 0: normal, < 10% reactive astrocytes, 1: mostly resting astrocytes and up to 30% reactive astrocytes, 2: some resting astrocytes and about 60% reactive astrocyte, 3: 100% reactive astrocytes.

### Morris water maze test

Following 60 days of severe hypoglycemia induction, 12 rats (Third batch, CG, *n* = 5; and HG, *n* = 7) were subjected to cognitive function assessments using the Morris water maze test.

The Morris water maze is a widely used test to assess spatial memory and learning in behavioral neuroscience. It was first introduced by Richard G. Morris in 1984 and is considered a gold standard in this field [20]. The test consists of a round water pool, approximately 6 feet in diameter and 3 feet deep, with a rescue platform in the center of the pool, a controlled water temperature of around 22 °C, and an animal behavior tracking system to monitor the path and other variables. Briefly, during the learning stage, the rats were subjected to 5 consecutive days of training as three trials/day. The animals were placed in the water pool for one minute each day to learn the location of the rescue platform. In this stage, the platform was higher than the water level. In the probe stage, which followed the learning stage, the rescue platform was removed, and the time and number of times the animals spent searching for the platform location were recorded. On the probe trial phase, three trials were conducted in a single day. The results of the the acquisition trials and probe trial phase in each day are presented as the average of the different trials.

### Statistical analysis

Statistical analyses were carried out using GraphPad Prism version 9.0 software (San Diego, California, USA). Repeated-measures analysis of variance (ANOVA) was employed to ascertain the significance of divergences in distance between the control and treatment groups throughout the acquisition phase. The t-test in conjunction with two-way ANOVA, succeeded by the least significant difference (LSD) post-hoc test, were used to discern group differences in diverse parameters between the two groups. The Mann-Whitney U test and the Kruskal-Wallis test were used for comparing ordinal variables. Experimental data are depicted as mean ± standard error of the mean (SEM), and statistical significance was set at *p* < 0.05.

## Results

### Physical and biochemical parameters

**Fig. 1:**
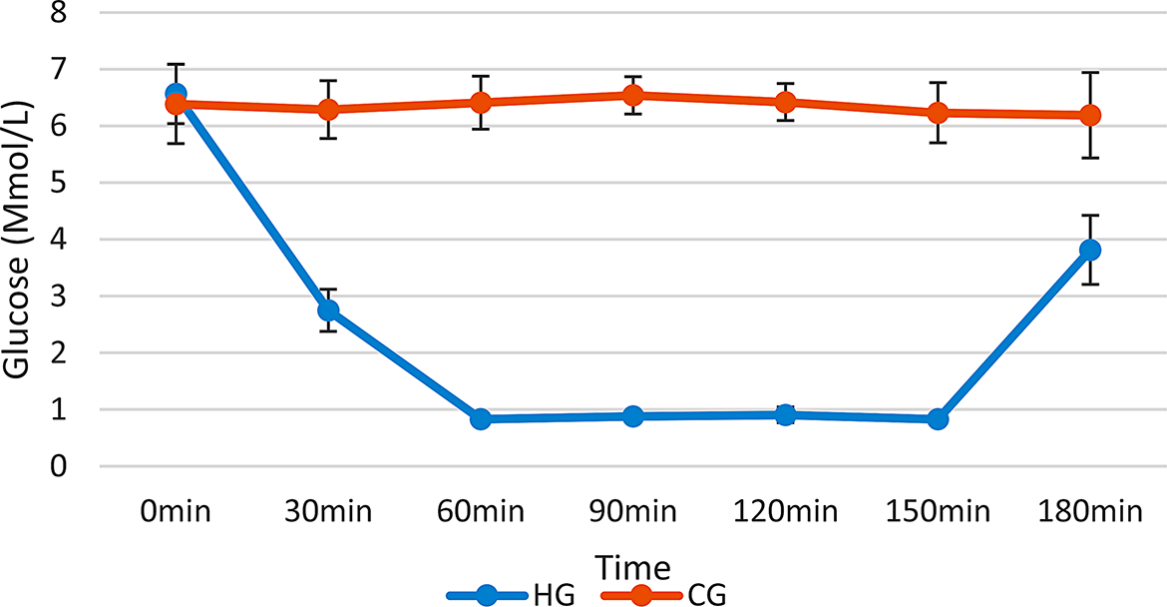
Glucose levels of the control and treatment groups during the severe hypoglycemia episode (Mean ± SD). CG: Control group, HG: Hypoglycemia group

During a severe episode of hypoglycemia, the glucose concentrations in the HG rats were carefully maintained within the range of 0.85-1 mmol/L for duration of 90 min, as illustrated in Fig. 1. Subsequently, all HG rats succumbed to a hypoglycemic coma within 90 min of the severe hypoglycemic event, exhibiting unresponsiveness to external stimuli and the loss of corneal reflex sensitivity. The hypoglycemic state was promptly alleviated through an intraperitoneal (i.p.) administration of a dextrose solution, followed by ad libitum access to food. Post-recovery assessments of blood glucose levels were conducted at one hour and 12 h post-termination of the hypoglycemic insult.

The overall mortality rate attributable to hypoglycemia was determined to be 34% (11 out of 32 rats), with fatalities occurring either during the acute hypoglycemic episode or within a 24-hour window subsequent to its induction. Among the 21 rats that underwent hypoglycemia and survived, 20 individuals (95.2%) experienced seizure activity in response to the hypoglycemic crisis.

Clinical manifestations including nervous tics, vocalization, and aggression were documented across all subjects. However, noteworthy improvement in these clinical symptoms was evident within three days following the cessation of hypoglycemic challenges, except for a minority of three rats (3/14, 21%) wherein such symptoms persisted over the entire 90-day monitoring duration.

### Effects of acute recurrent insulin-induced hypoglycemia on the stress oxidative biomarkers

**Fig. 2:**
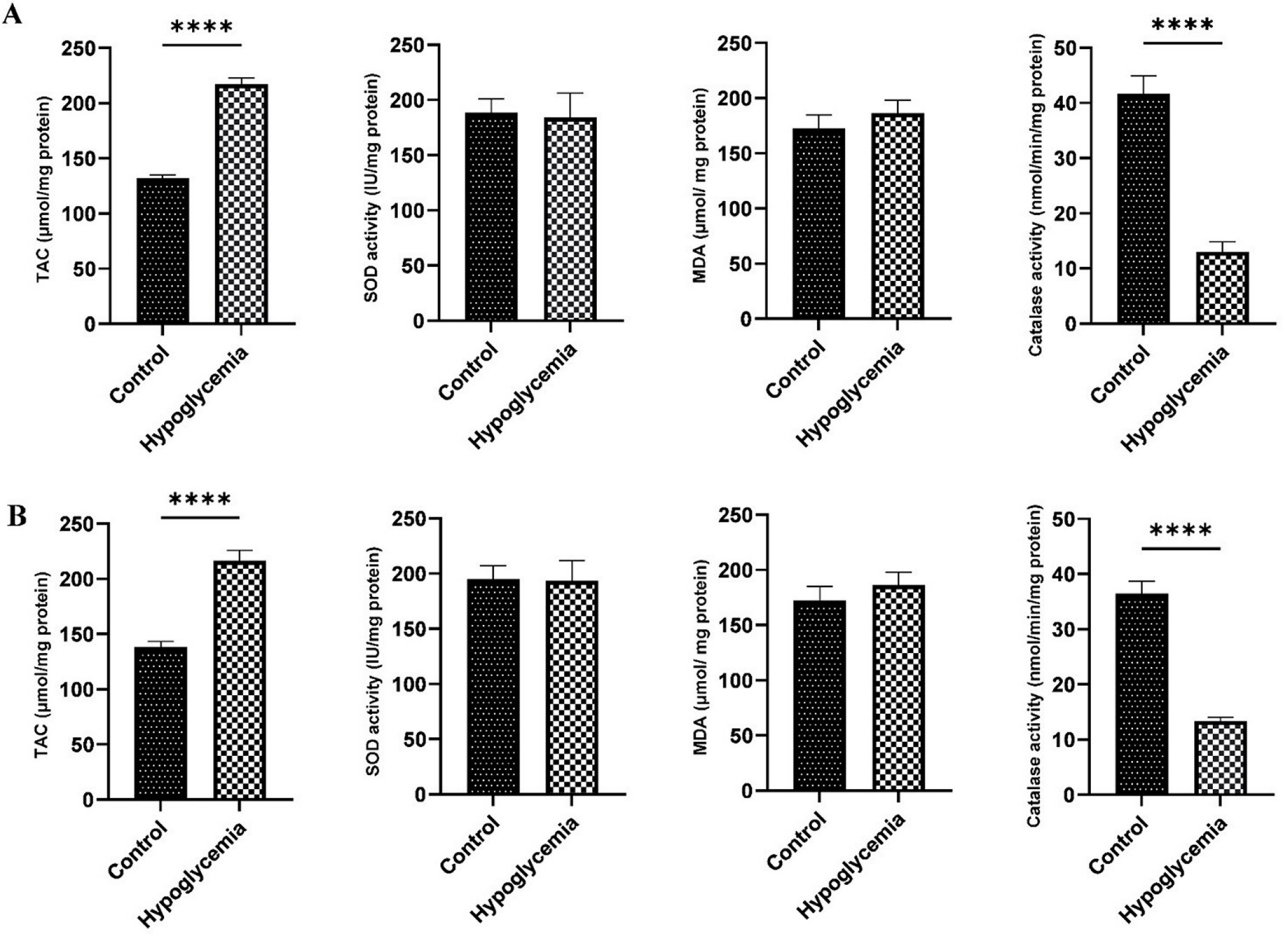
The effects of repeated episodes of severe hypoglycemia on oxidative stress (Mean ± SE) in the brain cortex (**A**) and hippocampal area (**B**). TAC: Total Antioxidant Capacity; SOD: Soperoxide Dismutase; MDA: Malondialdehyde. Compared to the control group, the levels of TAC increased and the activity of catalase decreased significantly in the hypoglycemic group both in the brain cortex and hippocampus. There was no significant difference in the levels of malondialdehyde and superoxide dismutase between the study groups. Significance levels are indicated by asterisks (****p < 0.0001)

Compared to the control group, the activity of catalase decreased significantly in the hypoglycemic group in both the brain cortex and hippocampus, while the levels of TAC increased. Interestingly, there were no significant differences noted in the levels of malondialdehyde and superoxide dismutase between the study groups (Fig. 2).

### Brain histology

**Fig. 3:**
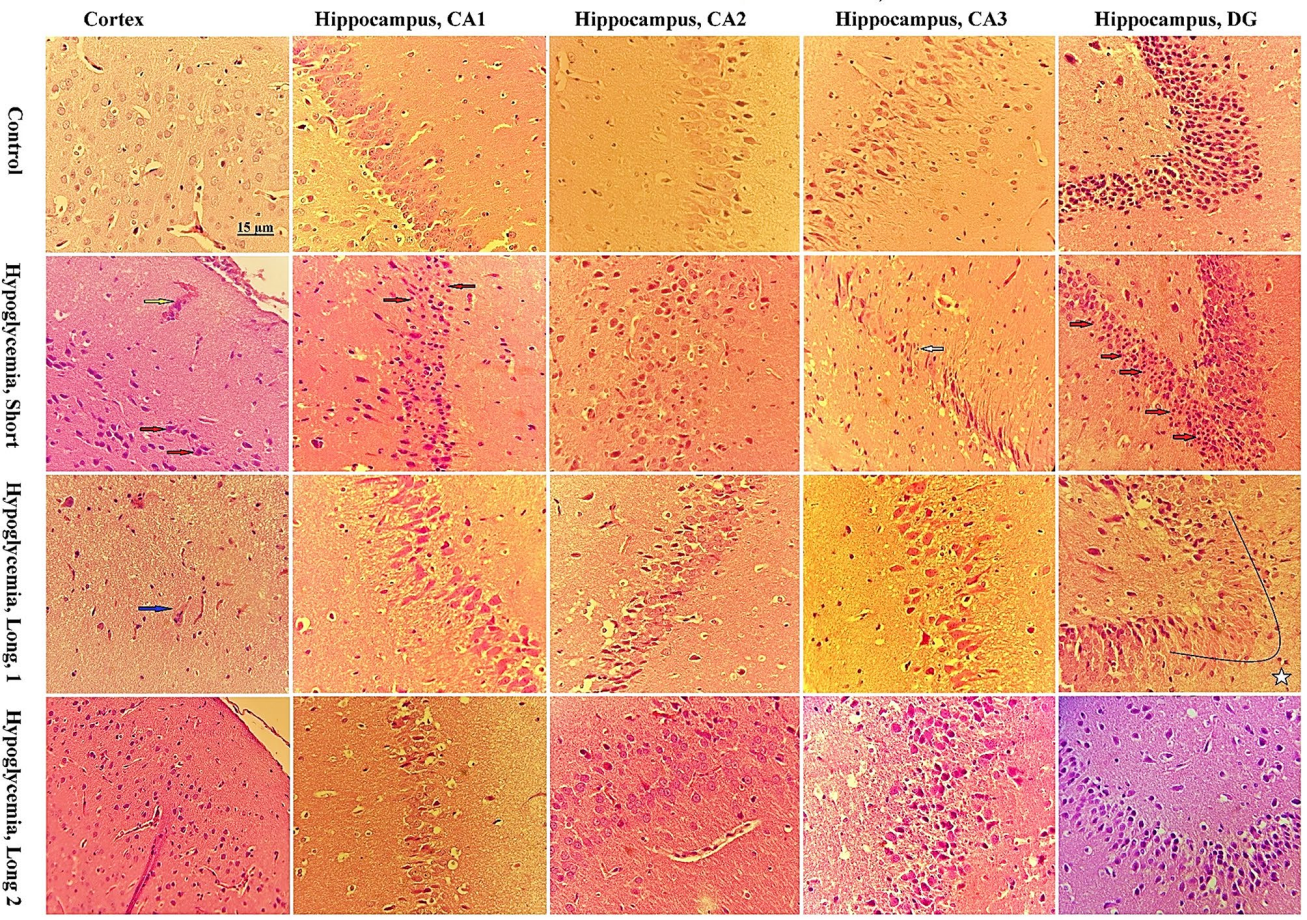
Photomicrographs of hematoxylin and eosin-stained brain cortex and hippocampus from control rats and rats subjected to 90 min hypoglycemia three times (400x magnification). Histopathological evaluation was performed at 7 days (Hypoglycemia, Short) and 3 months after induction of hypoglycemia. At 3 months after hypoglycemia induction, rats with neurological clinical signs (Hypoglycemia, Long1) were examined separately from rats without clinical signs (Hypoglycemia, Long2). In the evaluation of the cortex and hippocampus, no histopathological damage was observed in the control group. In the short-term evaluation, hyperemia (Yellow arrow) and pyknotic neurons with eosinophilic cytoplasm (Red arrow) were observed in the cortex area, and a significant number of pyknotic neurons (Red arrow) were observed in areas CA1 and DG areas of the hippocampus, and few neurons with karrhorexis nuclei (White arrow) were observed in CA3, and no significant damage was observed in CA2. In the long-term evaluation, in rats with clinical signs (Hypoglycemia, long1), a decrease in neuronal density, and presence of multi-nucleated neurons (Blue arrow) were observed in the cortex. The most significant changes observed in the hippocampus were a decrease in neuronal density in DG (Star). In the long-term evaluation of rats without clinical signs (Hypoglycemia, Long2), the most significant change observed was a mild decrease in neuronal density in DG, and no remarkable histopthology changes were seen in the cortex

To assess short and long-term histopathological changes following hypoglycemia induction, brain tissue samples were collected at 7 days and 90 days post-induction. Among the rats evaluated at the 90-day, 3 out of 7 exhibited persistent clinical symptoms in the form of nervous tics. Histopathological comparisons were made between this group and rats showing no clinical symptoms (Fig. 3). Each rat underwent histopathological assessment of the brain cortex and four parts of the hippocampus (CA1, CA2, CA3, and DG).

The histopathological evaluation conducted 7 days after hypoglycemia induction revealed varying degrees of changes in the cerebral cortex and hippocampus. Notable findings included numerous degenerating neurons, characterized as pyknotic, pink-red cells in the CA1 and DG regions, and with less intensity in CA3 region of the hippocampus, as well as petechial hemorrhages, neuropil rarefaction, and degenerated neurons in the cortex.

At the 90-day assessment post-hypoglycemia induction, rats displaying clinical symptoms showed reduced neuronal density, particularly in the DG regions of the hippocampus, and neuropil rarefaction in the cortex. Conversely, asymptomatic rats exhibited less severe histopathological changes, with a mild decrease in neuronal density in the DG without significant changs in the cortex.

**Fig. 4:**
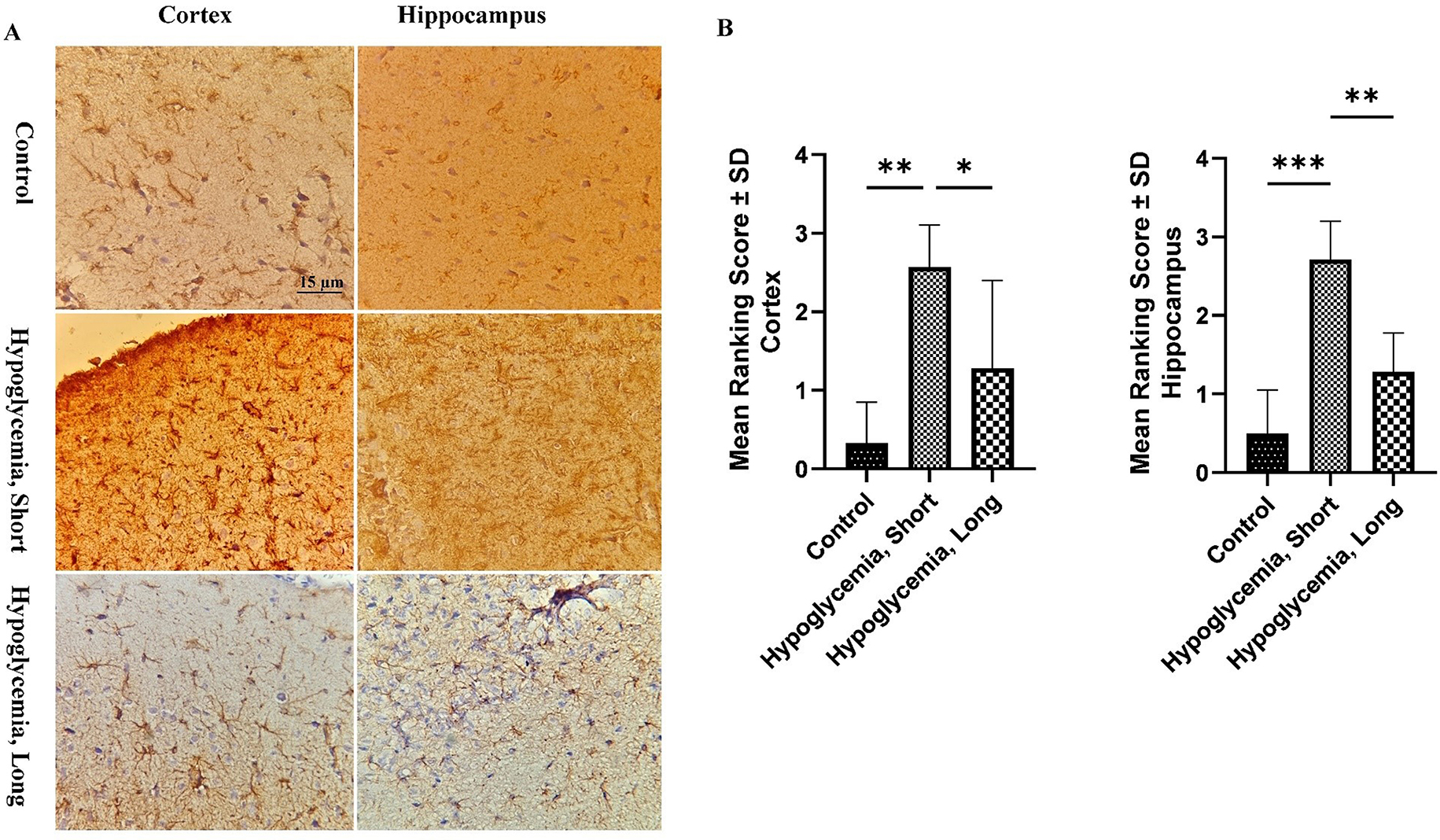
GFAP-positive astrocytes immunostained with rabbit anti‐GFAP polyclonal antibody (400x magnification). The impact of severe recurrent hypoglycemia on GFAP expression, serving as a marker for astrocyte development, has been demonstrated in both the brain cortex and hippocampus. Tissue samples were collected from the control group (10 rats) and from each treatment group (14 rats) at 7 days and 90 days after treatment. Three tissue sections of the cortex and three sections of the hippocampus were taken from each animal. The distance between the sections was 30 μm. (**A**) Representative immunohistochemical images showcasing GFAP expression in the brain and hippocampus of the control rats and rats subjected to 90 min hypoglycemia for three times, at 7 days (Hypoglycemia, Short) and 3 months (Hypoglycemia, Long) after induction of hypoglycemia (**B**). Semi-quantitative evalution of GFAP‐labeled astrocytes in the brain cortex and hippocampus. The results demonstrated rats subjected to hypoglycemia showed significant evidence of astrocytosis development. The severity of astrocytosis three months after inducing hypoglycemia (Hypoglycemia, Long) was significantly reduced compared to seven days after inducing hypoglycemia (Hypoglycemia, Short). Significance levels are indicated by asterisks (*p < 0.05, **p < 0.01, ***p < 0.001)

Astrocytes have key roles in many brain metabolic pathways, and reactive astrocytosis is a CNS response to all forms of brain injury and disorders [21]. Immunohistochemical staining for glial fibrillary acidic protein (GFAP) was conducted to visualize the reactive astrocytes in the brain tissue. GFAP immunostaining (Fig. 4) unveiled highly reactive astrocytes in rats subjected to recurrent severe hypoglycemia, displaying typical features such as dark enlarged cell bodies and thick processes distributed across the brain cortex and hippocampus. On a semiquantitative scale, the hypoglycemic rats showed a significant increase in GFAP-labeled astrocytes at both 7 and 90 days post-hypoglycemia compared to control rats. Interestingly, the severity of astrocytosis decreased three months after hypoglycemia induction compared to the initial one-week assessment.

### The results of morris water maze test

**Fig. 5:**
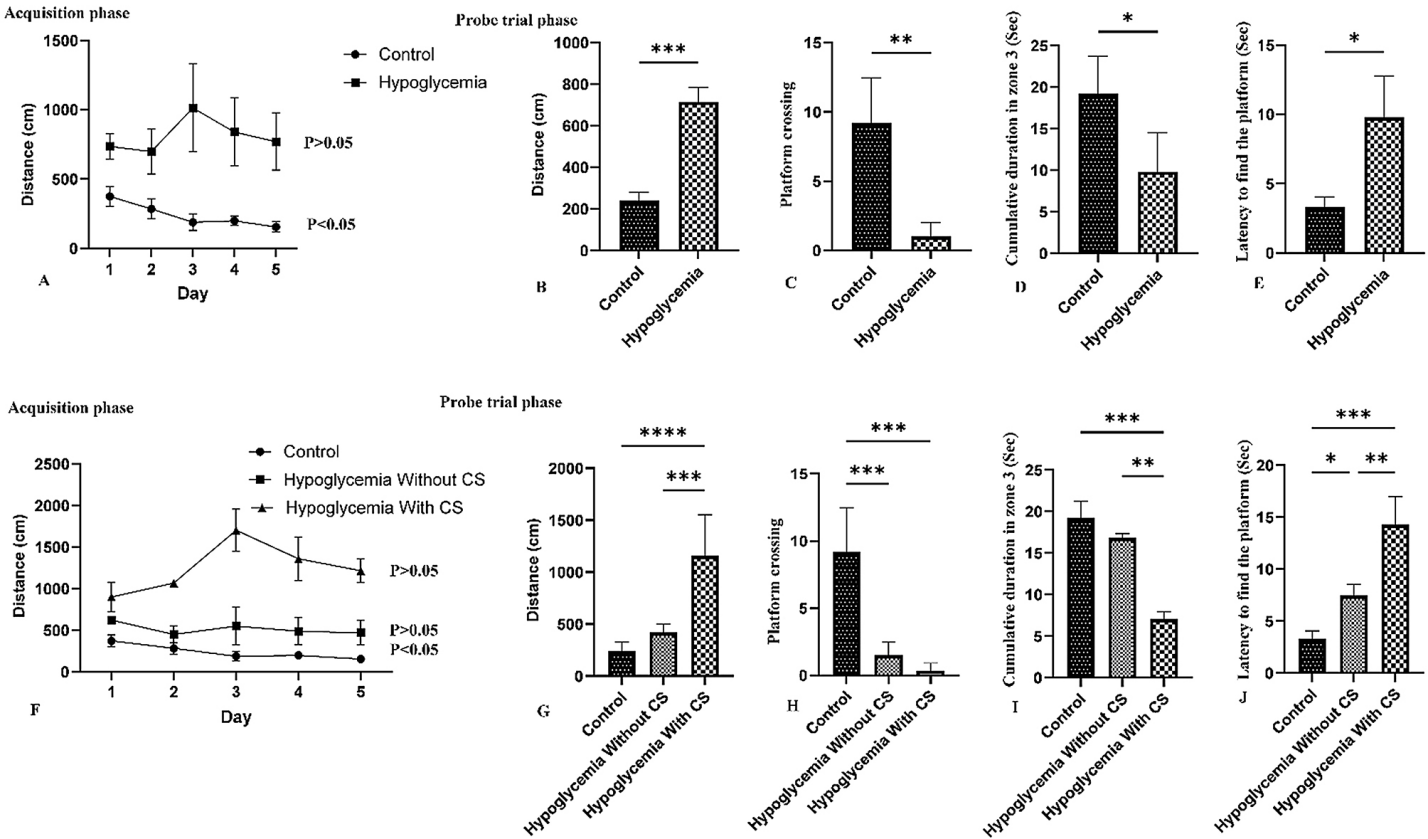
The results of Morris water maize in the control and hypoglycemic rats (Mean ± SE). The results of the Morris test were evaluated in two conditions. A comparison was made between the Morris test results in control rats and rats subjected to repeated episodes of severe hypoglycemia (**A**-**E**). Additionally, a comparison was made between the control group and rats that underwent repeated episodes of hypoglycemia with or without persistent clinical neurological signs (**F**-**J**). Statistical analysis of the Morris test data demonstrated that rats subjected to hypoglycemia showed significant evidence of cognitive dysfunction, with a greater severity of impairment observed in rats that displayed clinical signs. Significance levels are indicated by asterisks (*p < 0.05, **p < 0.01, ***p < 0.001, ****p < 0.0001)

The results of the Morris water maze tests were analyzed in two steps as illustrated in Fig. 5. Firstly, comparisons were made between the control and hypoglycemic groups. Subsequently, comparisons were made among the control group, hypoglycemic rats without clinical signs, and hypoglycemic rats with clinical signs.

During the acquisition trials, the spatial-memory training of the Morris water maze proved to be effective in the control group (*P* < 0.05, Fig. 5A). However, it did not show any significant differences in the distance traveled to discover the escape platform over five consecutive days in the hypoglycemic rats.

In the probe trial phase, significant differences were observed in distance, platform crossing, cumulative duration in zone 3, and latency to find the platform between the control and hypoglycemic groups. The statistical analysis revealed a significant difference in distance, cumulative duration in zone 3, and latency to find the platform between rats exhibiting clinical symptoms and those without clinical symptoms. Notably, no significant difference was found in distance and cumulative duration in zone 3 between the control group and rats without clinical symptoms.

## Discussion

Global diabetes prevalence has been on a steady rise worldwide. The number of individuals diagnosed with diabetes mellitus is projected to surge from 22.3 million in 2014 to 39.7 million by 2030 and further escalate to 60.6 million by 2060 [22]. Diabetes mellitus stands as a significant public health concern, ranking amongst the leading causes of mortality globally. Complications arising from diabetes afflict a majority of individuals with both type 1 and type 2 diabetes [23]. Neuropathy emerges as the most prevalent complication associated with diabetes [24]. In this context, not just hyperglycemia but also hypoglycemic episodes resulting from treatment play a significant role in neuropathy and neuronal damage. While some research indicates neurocognitive deficits in individuals experiencing severe hypoglycemia [25, 26], others suggest no long-term impairment in cognition. [27]

So, one of the primary goals of the present study was to investigate the impact of recurrent severe insulin-induced hypoglycemia on the cognitive deficits and the transient and permanent brain injuries in rat.

Young individuals with type 1 diabetes face a tenfold higher risk of sudden death compared to age-matched counterparts. Excess insulin administration resulting in severe hypoglycemia accounts for 6–10% of these fatalities [28, 29].

A study demonstrated that mortality as a result of severe hypoglycemia (glucose: 10–15 mg/dl) was 33% in non-diabetic rats [30]. In our current study, we observed a mortality rate of 34% in rats experiencing severe hypoglycemia, with deaths occurring as early as 30 min after the onset and persisting up to 24 h after the episode. The mechanisms underlying hypoglycemia-induced mortality remain inadequately understood. Brain neuroglycopenia, a pronounced sympathoadrenal response, and hypokalemia have been identified as potential triggers of fatal cardiac arrhythmias in patients experiencing severe hypoglycemia [30].

In diabetic patients, the risk of brain injury, cognitive dysfunction, and conditions like Alzheimer’s disease is elevated, with hypoglycemia likely playing a critical role in the development of cognitive impairment. The presence of significant brain edema in these patients indicates blood-brain barrier dysfunction, contributing to the progression of diabetic encephalopathy [31, 32]. Moreover, pericyte degeneration is thought to diminish oxygen supply to the brain, leading to neurovascular uncoupling, neuronal apoptosis, neuronal loss, and behavioral deficits in the cortex and hippocampus [33].

In our study, a short-term assessment conducted seven days post-hypoglycemia induction revealed significant histopathological changes primarily in the cortex, CA1, CA3, and DG regions of the hippocampus, characterized by hyperemia and neuronal apoptosis. Long-term evaluation at 90 days post-induction indicated a correlation between the intensity of histological changes in the rats and the severity of clinical symptoms observed. The histopathological changes in rats without neurological clinical symptoms were predominantly mild. The most noteworthy observation was a slight decrease in neuronal density in the DG, while no remarkable histopathological changes were evident in the cortex. However, in rats with persistent neurological clinical symptoms, the cerebral cortex exhibited multinucleated neurons and neuronal loss. Notably, the hippocampal evaluation revealed a significant loss of neurons in the DG region. It is plausible to infer that the hippocampus, particularly the DG region, is the most susceptible brain area to recurrent severe hypoglycemia, with the cortex following closely.

Hypoglycemia and increased blood insulin levels are both predisposing conditions to increase the formation of Amyloid β (Aβ) in the brain. Aβ is an aggregation-prone peptide, and intracellular accumulation of amyloid plaques may play an early role in Alzheimer’s disease pathogenesis [34]. Previous studies have shown that Aβ can interact with cellular prion protein (PrPC) and lead to increased cellular uptake. So the increased expression of PrPC significantly can enhance Aβ neurotoxicity and supports a role for PrPC in Alzheimer’s disease pathogenesis [35]. PrPC expression can be increased in hypoglycemia [36], and therefore, repeated periods of hypoglycemia in patients can play a crucial role in Alzheimer’s disease development, by higher PrPC-dependent uptake of Aβ. On the other hand, insulin shares the same degrading enzyme with Aβ as both insulin and Aβ are degraded by insulin-degrading enzyme and in insulin overdose, Aβ plaque formation can be induced through competition between insulin and Aβ for the insulin-degrading enzyme [37].

As previously mentioned, in the present study histopathological findings obtained three months post hypoglycemia induction revealed heightened hippocampal damage compared to the cortex. This suggests that even in the absence of clinical cortical symptoms, hypoglycemia development can significantly impair memory and learning abilities in patients. This concern extends beyond diabetic patients and likely impacts infants with neonatal hypoglycemia as well.

Neonatal hypoglycemia, characterized by low blood sugar levels in newborns, can profoundly affect brain function and development as glucose serves as the brain’s primary energy source. Preterm infants, those with low birth weight, and babies born to diabetic mothers commonly experience neonatal hypoglycemia, which can lead to brain injury and various cognitive and visual impairments, e.g., visual disturbances, occipital lobe epilepsy, cerebral palsy, and other associated complications [38].

Although neonatal hypoglycemia has been associated with severe complications, only a limited number of clinical follow-up studies have been conducted, leading to a lack of consensus on specific glucose thresholds for irreversible central nervous system damage. Recent research indicated that treating neonates with hypoglycemia below 1.7 mmol/L with glucose levels above 2.5 mmol/L did not significantly impact cognitive function or behavior [39].

In the current research, triggering hypoglycemia at a concentration of 1 mmol/L in rats led to more pronounced damage in the hippocampus than in the cortex. The Morris water maze results showed that this damage resulted in cognitive and memory impairments in the rats. The Morris water maze is a commonly employed behavioral test for evaluating spatial learning and memory in rodents [20]. However, various factors such as visual acuity, anxiety levels, and velocity can impact the animals’ performance in this test. To enhance the comparative analysis among patients, conducting an open field test alongside the Morris water maze test is suggested. Additionally, for a comprehensive evaluation of the neurological clinical symptoms associated with damage to different brain regions due to repeated severe hypoglycemic episodes, more extensive clinical assessments are necessary. In this study, initial assessments of visual acuity in rats were carried out using the optokinetic response test [40, 41] and the visual cliff test [42, 43].

Based on the findings from the Morris test in the current study, it was observed that hypoglycemia resulted in memory impairment in rats. However, the degree of impairment varied among the rats, with those exhibiting neurological symptoms experiencing more severe memory issues. Notably, significant hippocampal damage, especially in the DG region, was observed in these rats. Several studies have highlighted the connection between hypoglycemia and memory deficits. For instance, research by McNay et al. (2004) revealed that recurrent hypoglycemia led to cognitive impairments in rats, affecting spatial memory [44].

Additionally, research by Won et al. (2012) demonstrated that repeated hypoglycemic episodes resulted in cognitive decline and the thinning of the CA1 dendritic region six weeks post-hypoglycemia [45].

Rats that experienced a single episode of severe hypoglycemia showed condensed morphology and pyknotic nuclei in the cortex and hippocampus, specifically in the CA1, CA3, and DG, however, the severity of the damage was greater in regions CA1 and DG [46]. The CA1 region in the hippocampus is involved in spatial memory processing [47], and the DG region in the hippocampus plays a crucial role in neurogenesis and cognitive function [48].

Multiple factors are involved in the development of hypoglycemia-induced brain injury, such as oxidative stress, heightened production of reactive oxygen species, elevated levels of inflammatory factors like TNF-α and IL-6, and the activation of MMP9. These factors can degrade various structural components of the extracellular matrix and non-extracellular matrix proteins, playing pivotal roles in hypoglycemia-related brain damage [49, 50]. Based on the literature review, it appears that insulin possesses both oxidant and antioxidant effects. A recent study has demonstrated that insulin, through the Nrf2 signaling pathway, could increase some antioxidant proteins and prevents neurodegenerative damages associated with diabetes mellitus [51]. Furthermore, research indicates that administering insulin at a dosage lower than the body’s requirement can potentially reduce inflammatory cytokines and the oxidative stress response, thereby potentially enhancing the improvement of brain tissue damage. [15], whereas higher-than-necessary doses may lead to adverse effects. Research indicates that high levels of insulin result in increased NADPH oxidase 4 (NOX4) activity, consequently leading to higher production of reactive oxygen species [52]. In the current research, it was observed that after repeated episodes of insulin-induced severe hypoglycemia in rats, there was a significant decrease in the brain tissue level of catalase. Interestingly, despite this decrease, the total antioxidant capacity of the brain actually increased in rats. However, neurodegenerative damages resulting from hypoglycemia were observed in rats. These findings suggest that oxidative stress probably does not play a fundamental role in the pathogenesis of neurodegenerative damages in cases of severe insulin-induced hypoglycemia. The most important mechanism involved in neuronal damages following hypoglycemia is the excitotoxicity caused by several-fold elevations in brain extracellular glutamate and aspartate concentrations. Other mentioned mechanisms of neurodegeneration in these patients include reduced protein synthesis, decreased energy, loss of ion homeostasis, cellular calcium influx, and cellular alkalosis [14, 53]. In the present study, a notable decrease in catalase activity was observed in the hypoglycemic group, both in the brain cortex and hippocampus. Catalase, an essential antioxidant enzyme, plays a pivotal role in safeguarding the brain against oxidative damage during hypoglycemic episodes. Previous animal research has demonstrated that the upregulation of catalase even in the absence of alteration in markers of oxidative stress can enhance cognition and reduce measures of anxiety. The authors have suggested that these findings may be due to altered redox signaling, which have resulted from catalase overexpression [54]. Catalase has also been implicated in the pathogenesis of neurodegenerative diseases such as Alzheimer’s and Parkinson’s disease. Studies have reported decreased catalase activity in the brains of patients with Alzheimer’s and Parkinson’s disease, suggesting a role for oxidative stress in the development of these diseases [55]. Moreover, animal studies have shown that overexpression of catalase can improve cognitive function and reduce oxidative stress in models of Alzheimer’s and Parkinson’s disease [56]. In the current study, it is likely that the reduction of catalase activity has played a remarkable role in the pathogenesis of brain injuries and cognitive deficits in rats subjected to recurrent severe hypoglycemia. However, considering the increase in total antioxidant capacity and the lack of significant results in the levels of MDA and SOD, it does not seem that oxidative stress plays a fundamental role in neurodegenerative injuries in rats subjected to severe insulin-induced hypoglycemia.

## Conclusion

Severe recurrent episodes of hypoglycemia can cause significant cognitive impairment and brain damage, especially affecting the hippocampus. 21% of surviving subjects exhibited persistent neurological symptoms, while all rats displayed cognitive dysfunction. This issue is probably not limited to diabetic patients; it may also be a major factor in brain damage and impaired learning abilities in cases of neonatal hypoglycemia. It does not appear that oxidative stress plays a central role in neuronal damage following severe insulin-induced hypoglycemia. Nevertheless, more research is necessary to assess the impact of repetitive hypoglycemic episodes on enduring damage in various brain regions. The severity, duration of hypoglycemia, and individual characteristics all play key roles in determining the extent of resulting damage. Moreover, further exploration is crucial to crafting effective prevention and treatment strategies for hypoglycemia-induced brain damage and neurodegenerative conditions.

### Electronic supplementary material

Below is the link to the electronic supplementary material.

Supplementary Material 1

## Data Availability

The datasets used and/or analyzed during the current study are available from the corresponding author on reasonable request.
