# Supplementary material for: Effect of recurrent severe insulin-induced hypoglycemia on the cognitive function and brain oxidative status in the rats
Source: Diabetol Metab Syndr. 2024 Jul 15;16:161. doi: 10.1186/s13098-024-01410-z (PMC11247731; doi:10.1186/s13098-024-01410-z)
Supplement: Supplementary file 1 — Supplementary Material 1 [file 13098_2024_1410_MOESM1_ESM.docx]

Glucose values in the hypoglycemic group during and after hypoglycemia induction

Mean (mg/dl):

| 118.142857 | 49.4761905 | 14.952381 | 15.8095238 | 16.3333333 | 14.9047619 | 68.6666667 |
| --- | --- | --- | --- | --- | --- | --- |

SD:

Glucose values in the hypoglycemic group during and after hypoglycemia induction

Mean (Mmol/L):

| 6.56349206 | 2.74867725 | 0.83068783 | 0.87830688 | 0.90740741 | 0.82804233 | 3.814815 |
| --- | --- | --- | --- | --- | --- | --- |

SD:

|  | |  | |  | |  | |  | |  | |  | |
| --- | --- | --- | --- | --- | --- | --- | --- | --- | --- | --- | --- | --- | --- |
| 0.52331025 | 0.37169077 | | 0.07753173 | | 0.0835535 | | 0.13870358 | | 0.08941642 | | 0.608919 | |  |

Glucose values in the Control group during and after Normal saline administration

Mean (mg/dl):

| 115 | 113.2 | 115.4 | 117.7 | 115.6 | 112.2 | 111.4 |
| --- | --- | --- | --- | --- | --- | --- |

SD:

| 12.58747 | 9.174845 | 8.395766 | 5.9264 | 5.872724 | 9.554522 | 13.54991 |
| --- | --- | --- | --- | --- | --- | --- |

Glucose values in the Control group during and after Normal saline administration

Mean (Mmol/L):

| 6.388889 | 6.288889 | 6.411111 | 6.538889 | 6.422222 | 6.233333 | 6.188889 |
| --- | --- | --- | --- | --- | --- | --- |

SD:

| 0.699304 | 0.509714 | 0.466431 | 0.329244 | 0.326262 | 0.530807 | 0.752773 |
| --- | --- | --- | --- | --- | --- | --- |

Total values
